# Cost-effectiveness of dexamethasone compared with aflibercept in naïve diabetic macular edema

**DOI:** 10.1186/s12962-022-00401-z

**Published:** 2022-12-01

**Authors:** Paula Montes Rodríguez, Javier Mateo Gabás, Olivia Esteban Floría, Ana Honrubia Grijalbo, Francisco J. Ascaso Puyuelo

**Affiliations:** 1 Department of Ophthalmology, Hospital Clínico Universitario “Lozano Blesa’’, Zaragoza, Spain; 2 Aragon Health Research Institute (IIS Aragon), Zaragoza, Spain

**Keywords:** Diabetic macular edema (DME), Cost-effectiveness, Cost-utility, Aflibercept, Delayed-release device of dexamethasone

## Abstract

**Background:**

To assess the cost-effectiveness of the delayed-release device of dexamethasone compared with aflibercept in the treatment of patients with naïve diabetic macular edema (DME) from a societal perspective in the healthcare sector Zaragoza III in Spain.

**Methods:**

A Markov model with five states defined by visual acuity (VA) in the better-seeing eye (Snellen scale) and an additional death state were constructed. Two cohorts of patients were distributed along the VA states and treated during a year with either dexamethasone or aflibercept. One-year follow-up on each group was performed. Medical costs related to the DME treatment and follow-up, medical costs related to the DME comorbidities, and non-medical-related costs were taken into account. Costs (2020 €), health outcomes (Quality-Adjusted Life Years-QALYs), both discounted at a 3.5% annual rate, and incremental cost-effectiveness ratios (ICER: €/QALY) were determined for a lifetime horizon in the base case analysis.

**Results:**

Patients treated with dexamethasone were €77,349 more costly and provided 2.667 additional QALYs (€29,002/QALY) than those treated with aflibercept. The variable efficiency per patient was calculated dividing the improvement in quality of life (on the VFQ-25 scale) by the cost of the treatment. With the obtained results it can be concluded that the efficiency of treating the patients with dexamethasone is significantly superior than the efficiency of treating them with aflibercept.

**Conclusions:**

The cost per QALY gained with the delayed-release device of dexamethasone compared with the one obtained by aflibercept in the naïve DME population is just below the €30,000 threshold, below which, new drugs are sometimes regarded as cost-effective strategies in Spain. In this model, the key variables with greater impact on the cost-effectiveness results were the selected time horizon, the chosen extrapolation method and the number of aflibercept and dexamethasone injections.

## Background

According to the World Health Organization, diabetes is currently the leading cause of blindness in the working population in developed countries; and in Spain, it affects 6–18% of its population [1]. The main reason for loss of vision in naïve diabetic patients is due to the macular edema, present in approximately 5.5% of them [1]. The Wisconsin Epidemiologic Study of Diabetic Retinopathy found that 20% of patients with type 1 diabetes and 25% of those with type 2 diabetes will develop DME after 10 years of follow-up [2].

This study utilized existing guidelines to examine cost-effectiveness of the two accepted and most widely used interventions for DME treatment [1, 3].

The high cost of newer therapies against DME has brought the attention of ophthalmologists to the field of pharmacoeconomics.

Since June 2018, the protocol of the Aragon Drug Evaluation Commission (CEMA) has been applied for the treatment of DME in Aragon [4]. In this protocol, aflibercept (Eylea®), as an antiangiogenic drug, and the delayed-release device of dexamethasone (Ozurdex®), as a corticosteroid, are considered the first-line treatment for this pathology.

Few cost-effectiveness studies have been carried out in relation to DME treatment, with; almost all of them outside Spain and none in Aragon. We consider that, given the importance of the pathology being studied, the high cost generated by its treatment and the disability it causes, it would be very useful to carry out a cost-effectiveness study.

In the present study we sought to examine the cost-effectiveness of dexamethasone compared to aflibercept in the naïve DME population [3] to determine which is more cost-effective.

## Methods

### Study design

**Fig. 1:**
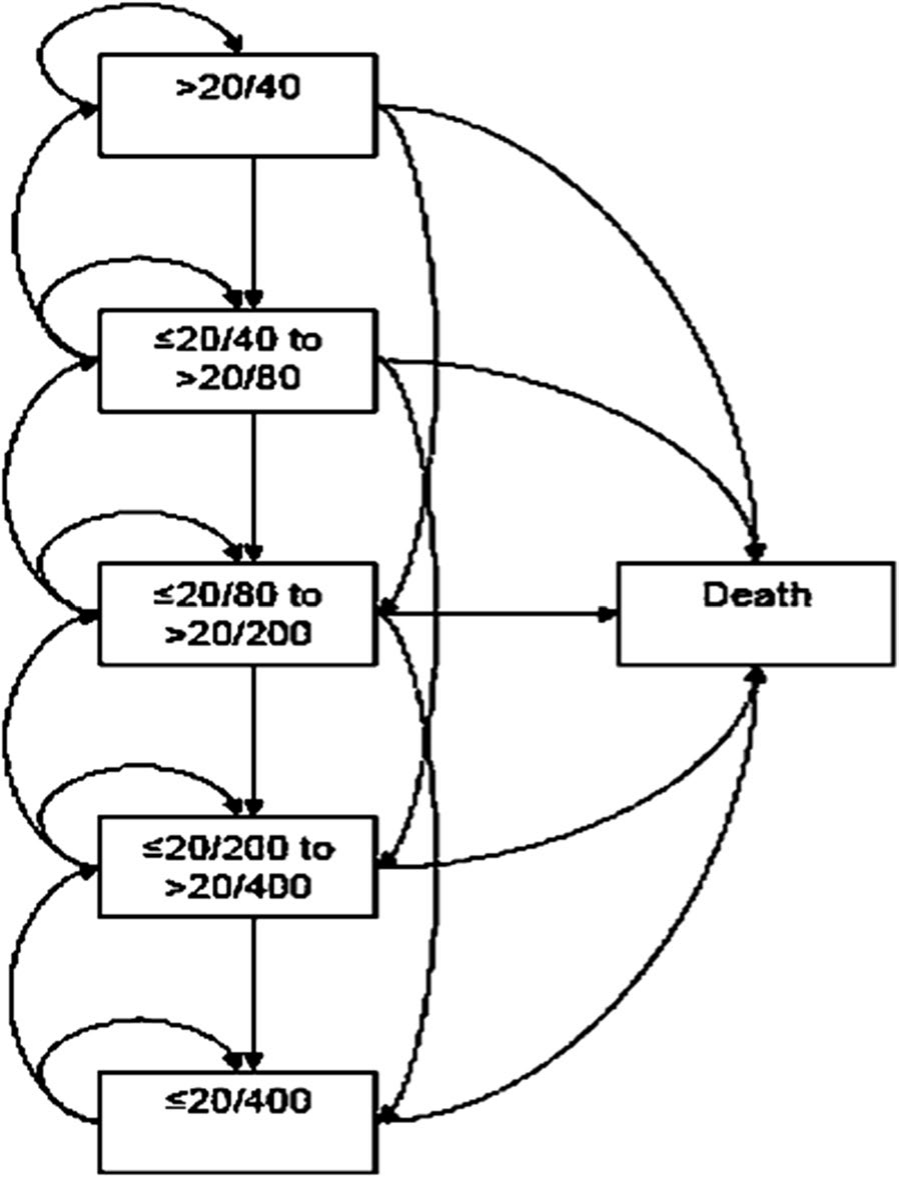
Markov model with the five sates defined by visual acuity in the better-seeing eye, and an additional death state. Arrows indicate allowed transitions

A Markov model with five states was constructed and defined by the visual acuity (VA) in the better-seeing eye: better than 20/40 in the Snellen scale, ≤ 20/40 to > 20/80, ≤ 20/80 to > 20/200, ≤ 20/ 200 to > 20/400, ≤ 20/400, and an additional death state. We present the model scheme with its transitions in Fig. 1. Markov models are usually used in the field of decision analysis to model the progression of chronic diseases. By attaching estimates of resource use and health outcomes to the states in the model, it is possible to estimate the long-term costs and outcomes associated with different healthcare interventions [5]. In the context of economic evaluations, models provide the appropriate framework to synthesize all available evidence, compare all relevant treatment options, and systematically study the impact of different scenarios and assumptions through sensitivity analysis [6].

The study was carried out in the field of healthcare activity and routine practice at the Ophthalmology Service of Hospital Clínico Universitario "Lozano Blesa" in Zaragoza. All naïve diabetic patients from Zaragoza III health sector were recruited to participate in the study and they were followed-up for a year. The cohort of patients was distributed along the five VA states at the time of initiation of treatment, according to data from a Spanish study [7] and randomized to the aflibercept 40 mg/ml solution for injection treatment group or the dexamethasone delayed-release device 700 μg in an applicator (Ozurdex®) treatment group. The main cohort included 134 patients with type 2 diabetes and clinically significant DME. 77 (57%) were male and 57 (43%) female and the mean age was 68.1 years old. All patients had prior cataract surgery and had not previously received DME treatment. These features were similar to those of the basal populations of the main DME clinical trials.

The transition probabilities for dexamethasone were obtained from efficacy results of the Schwartz et al. trial [8] and transition probabilities between VA states for patients treated with aflibercept were obtained from the Vivid-east study [9]. The transition probabilities to the “death” state were taken from Spanish life tables [10]. A 3-month cycle length was selected. One-year probabilities obtained from clinical trials were transformed into 3-month probabilities with the formula p = 1–e^−rt^ [11]. The model was constructed and solved with the TreeAge Pro Suite 2008 software package (TreeAge Software, Williamstown, MA, USA).

### Perspective and costs

This study was conducted, from the societal perspective, in a Spanish setting. Costs are presented in 2020 €. The following costs were taken into account: direct medical costs related to the DME treatment and follow-up (i.e. drugs, physicians’ honoraries, diagnostic procedures, adverse reactions, vision rehabilitation related costs and vision-enhancing equipment related costs), direct medical costs related to the DME comorbidities (i.e. fall/accidents, depression/anxiety and other conditions requiring medical treatment) and non-medical-related costs (i.e. assistance from paid professionals for daily activities and social benefits received for visual disabilities).

Medical costs related to the DME comorbidities and non-medical-related costs were obtained from a multi-country, cross-sectional, observational study in which information was gathered directly from patients with bilateral DME and compared to control subjects [12].

With regard to medical costs related to the DME treatment, costs for physician consultations and diagnostic procedures were obtained from the previous study [12]. Resource use was determined by a retina specialist. To calculate the drug costs, aflibercept and dexamethasone vial prices were taken from the Spanish Council of Pharmacists database, and then multiplied according to the number of injections performed in the one-year follow-up. Costs derived from adverse reactions were calculated by multiplying endophthalmitis, lens damage and retinal detachment trial rates by the cost of the diagnosis-related group in Spain [13]. We also included mean annual per-patient vision rehabilitation related costs and vision-enhancing related costs which accounted for €69 and €211 respectively [13]. Table 1 depicts previously described.

**Table 1:** Direct medical costs related to DME treatment and follow-up (€) for dexamethasone and aflibercept

	Unit cost mean^a^ (SD) Euros	Annual resource use^b^	
		Aflibercept	Dexamethasone
Retina specialist consultation	112 (24)	12 (6–18)	8 (4–12)
Fundus photography	20 (4)	12 (6–18)	8 (4–12)
Optical coherence tomography	149 (30)	6 (3–9)	4 (2–8)
Fluorescein angiography	42 (8)	2 (1–3)	2 (1–3)
Dexamethasone vial	750.92 (208)	–	3.1^c^
Aflibercept vial	588.8 (139)	9.8^c^	–
Adverse reactions^d^			
Endophthalmitis	3156 (631)	0.006	0.0128
Lens damage	1600 (320)	0.004	0.006
Retinal detachment	3702 (740)	0.004	0.007

^a^Unit costs were obtained from an observational study [10] and inflated to 2020 € with Spanish health care indices. Unit costs for drug vials were obtained from the Spanish Council of Pharmacists Database. Aflibercept and Dexamethasone are 100% reimbursed by the Spanish National Health System. SDs (standard deviation) were selected to produce variation coefficients of 20%

^b^The number of follow on consultations and diagnostic procedures per patient/year were determined by a retina ophthalmologist. In parenthesis, the range of values tested in the probabilistic sensitivity analysis is provided. The number of drug injections and adverse reactions per patient/year were obtained from the VISION and VISTA trials

^c^The number of per-year aflibercept and dexamethasone injections were tested in the sensitivity analysis

^d^Unit costs for the diagnosis-related group in Spain were obtained from the Spanish Health Ministry

Total mean annual costs per patient were estimated to be €771 and €1577 respectively. These costs were compared across VA levels in the better-seeing eye, and the observed differences did not reach statistical significance [12], thus we used the same figure in the model for each VA state.

Indirect costs (i.e. costs related to productivity loss) were not included in the base-case analysis. However, it is unlikely that many people of this age are employed. The impact of including indirect costs derived from productivity loss by family members is explored in the sensitivity analysis.

Health care costs were inflated to Euro 2020 with specific Spanish health care price indices. Non-medical costs were inflated with general consumer price indices [14].

### Time horizon

Patients made five clinical visits in both study arms. A loading dose of three consecutive monthly injections was applied in the aflibercept treatment group, following a PRN treatment guideline. In the delayed-release device for dexamethasone treatment group one injection was scheduled and then also followed the PRN treatment guideline. Then a life-expectancy time horizon was selected in the base-case analysis because the effect of treating with both drugs was not restricted to a limited number of months in clinical practice. Several approaches for extrapolating clinical trial data over the entire lifetime horizon were explored. Survival probabilities according to the patient’s age were obtained from Spanish life tables [10].

### Utilities

Medical interventions can improve either survival or quality of life, or both. Ophthalmologic treatments directed towards DME can improve quality of life by means of improving VA. Cost-utility analyses are economic evaluations in which quality of life is taken into account. The Quality Adjusted Life Year (QALY) is usually the health outcome measure used in cost-utility analyses. QALYs are calculated by multiplying years spent in a certain health state by a factor—utility—that quantifies preference for that health state. By convention, utilities vary from 1.0 (perfect health) to 0.0 (death). Several techniques for obtaining individuals’ preferences for health outcomes are available. The measurement task can be bypassed using a pre-scored multi-attribute health-status classification system. Among the general health systems, the most widely used is the Health Utilities Index (HUI) or the Visual Function Questionnaire (VFQ-25). Detailed explanations of these methods are available elsewhere [15]. However, it is important to remark that the choice of one method over another has an impact on the health utilities obtained. Thus, in economic evaluations it is important to mention the chosen instrument, and perform sensitivity analysis when different data are available. In this study, published utilities obtained from a cohort of DME patients with the time trade-off methodology were used (Table 2) [16]. Utilities correlated with VA in the better-seeing eye [16, 17]. Participant VA levels at each visit were converted to QALYs using data from Brown et al. [16], who linked VA in a patient’s better-seeing eye with health-related quality of life. The VAs were obtained from the trial, converted to Snellen acuities, and assigned a utility based on conversion tables [16]. Quality-of-life levels at monthly visits during the first year were summed, providing an aggregate QALY value for the entire year for each participant.

**Table 2:** Utilities for each visual acuity state

Visual acuity	Time trade-off	Standard gamble
> 20/40	0.85 (0.75–0.95)	0.90 (0.83–0.97)
≤ 20/40 to > 20/80	0.78 (0.72–0.84)	0.92 (0.88–0.96)
≤ 20/80 to > 20/200	0.78 (0.67–0.89)	0.84 (0.72–0.96)
≤ 20/200 to > 20/400	0.64 (0.53–0.75)	0.71 (0.58–0.84)
≤ 20/400	0.59 (0.23–0.95)	0.70 (0.29–1.11)

Utilities according to visual acuity in the better-seeing eye obtained in a cohort of patients with DME [16]

### Discount

In order to obtain the net present value of future costs and health outcomes accruing over the entire time horizon, a discounting rate of 3.5% was applied to both, as it was recommended by the National Institute for Clinical Excellence guidelines [18].

### Sensitivity analysis

The following parameters were included in the probabilistic sensitivity analysis: costs, transition probabilities, VA state utilities assigned by patients and resource use (i.e. number of fundus photography, optical coherence tomography, fluorescein angiography, and ophthalmologist consultations). Probability distributions were chosen for each parameter according to the published recommendations [19]. For transition probabilities (Table 3) and patients’ utilities (Table 2) which range from 0 to 1, a beta distribution was selected. The parameters α and β in the beta distributions were approximated using mean and SD values obtained from published utilities [16]. A gamma distribution, which is positively skewed, was selected for costs. In order to account for variability in costs, SDs were selected that produced variation coefficients of 20% for unit costs of diagnostic procedures and consultations (Table 1), as well as costs related to DME comorbidities and non-medical costs [12]. A uniform distribution was selected for resource use (Table 1).

**Table 3:** Three-month transition probabilities between visual acuity states for dexamethasone and aflibercept

Visual acuity	> 20/40	≤ 20/40 to > 20/80	≤ 20/80 to > 20/200	≤ 20/200 to > 20/400	≤ 20/400
Dexamethasone					
> 20/40	0.9863	0.0107	0.0030	0	0
≤ 20/40 to > 20/80	0.0979	0.8884	0.0107	0.0030	0
≤ 20/80 to > 20/200	0	0.0979	0.8884	0.0107	0.0030
≤ 20/200 to > 20/400	0	0	0.0979	0.8884	0.0137
≤ 20/400	0	0	0	0.0979	0.9021
Aflibercept					
> 20/40	0.9199	0.0542	0.0259	0	0
≤ 20/40 to > 20/80	0.0153	0.9046	0.0542	0.0259	0
≤ 20/80 to > 20/200	0	0.0153	0.9046	0.0542	0.0259
≤ 20/200 to > 20/400	0	0	0.0153	0.9046	0.0801
≤ 20/400	0	0	0	0.0153	0.9847

As it was aforementioned, several approaches were explored for extrapolation beyond the one-year follow-up duration of the study. Under the ‘continuous treatment effect’ approach selected for the reference case, both treatments are performed, each with efficacy lasting over the whole time horizon [11]. In addition to the ‘continuous treatment effect’ approach, we tested both a ‘one-time benefit’ approach and a ‘rebound’ or ‘catch-up’ approach [11]. Under the ‘one-time benefit’ approach, both treatments stop after one year. From then on, patients’ quality of life declines at the same rate for both drugs. Additional QALYs are therefore gained by projecting the area under the curve over a longer period.

On the other hand, we also explored costs and health outcomes in the shorter one-year time horizon. This short-time horizon represents a worst-case scenario for dexamethasone because clinical benefit is assumed to last for 6 months only.

A sensitivity analysis using the number of administrations from our study was carried out (three fixed monthly doses scheduled for aflibercept and two injections for dexamethasone). After three consecutive monthly intravitreal injections, further retreatment was performed on an “as needed” basis.

### Model validation

Thorough internal testing of the model was performed, and the expected outcomes were obtained when different input values were used. The model could not be calibrated against external data, due to the absence of data over the time frame being modelled.

Therefore, different assumptions in the sensitivity analyses were explored in order to model long-term effectiveness data.

### Outcomes

Costs (Euro 2020), health outcomes (QALYs) for both aflibercept and dexamethasone, and incremental cost-effectiveness ratios (ICER; €/QALY) were obtained for the base-case analysis and for those alternative scenarios considered in the sensitivity analyses.

### Results

In the base-case analysis (Table 4), treating patients with naïve DME with dexamethasone instead of aflibercept is €77,349 more expensive, and provides 2.667 more QALYs in the lifetime horizon, thus providing an ICER of €29,002/QALY.

**Table 4:** Results for the reference case

	Cost (€)	Incremental cost (€)	Efficacy (QALYs)	Incremental efficacy (QALYs)	ICER (€/QALY)
Aflibercept	92,340	–	4.134	–	–
Dexamethasone	169,689	77,349	6.80	2.667	29.002

ICER: incremental cost-effectiveness ratio. QALY: quality-adjusted life year. Costs are presented in 2020 Euro

**Fig. 2:**
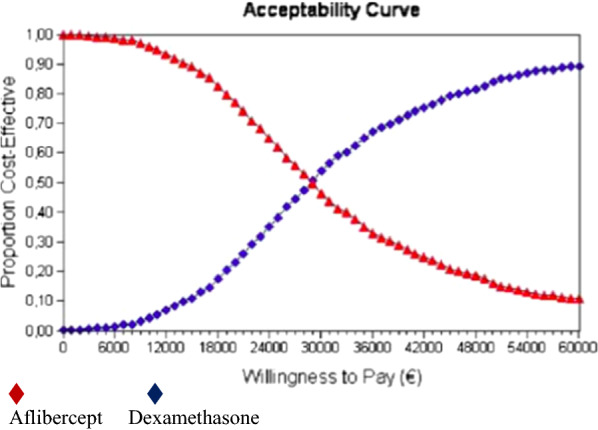
Aflibercept (red). Dexamethasone (blue). Acceptability curve obtained with probabilistic sensitivity analysis in the lifetime horizon. Below the 30.000 €/QALY, dexamethasone is the therapy of choice in 54% of cases

According to the probabilistic sensitivity analysis, dexamethasone was the therapy of choice in 54% of cases below the threshold of €30,000/QALY in the lifetime horizon (Fig. 2).

Alternative sensitivity analyses results are depicted in Table 5.

**Table 5:** Results of the sensitivity analysis

Parameter/variable	ICER
Reference case	29,002
No extrapolation (2-year time horizon)	119,953
Extrapolation beyond 2 years	
One-time benefit approach	32,795
Beyond approach	52,031
Injections taken from the PrONTO trial	4623
2-year time horizon	20,472
Efficacy for aflibercept from naive patients	14,302
Starting age	
58 years old	24,553
90 years old	51,798
Starting VA stage	
> 20/40	33,653
≤ 20/40 to > 20/80	27,855
≤ 20/80 to > 20/200	26,268
≤ 20/200 to > 20/400	29,874
≤ 20/400	36,372
Method for utility elicitation	36,186
Discounting rate	
0%	26,990
5%	30,247
One-year cycle length	30,642
Including indirect cost derived from family members time to assist patients	26,900
Efficacy for dexamethasone from naive patients	85,300

**Fig. 3:**
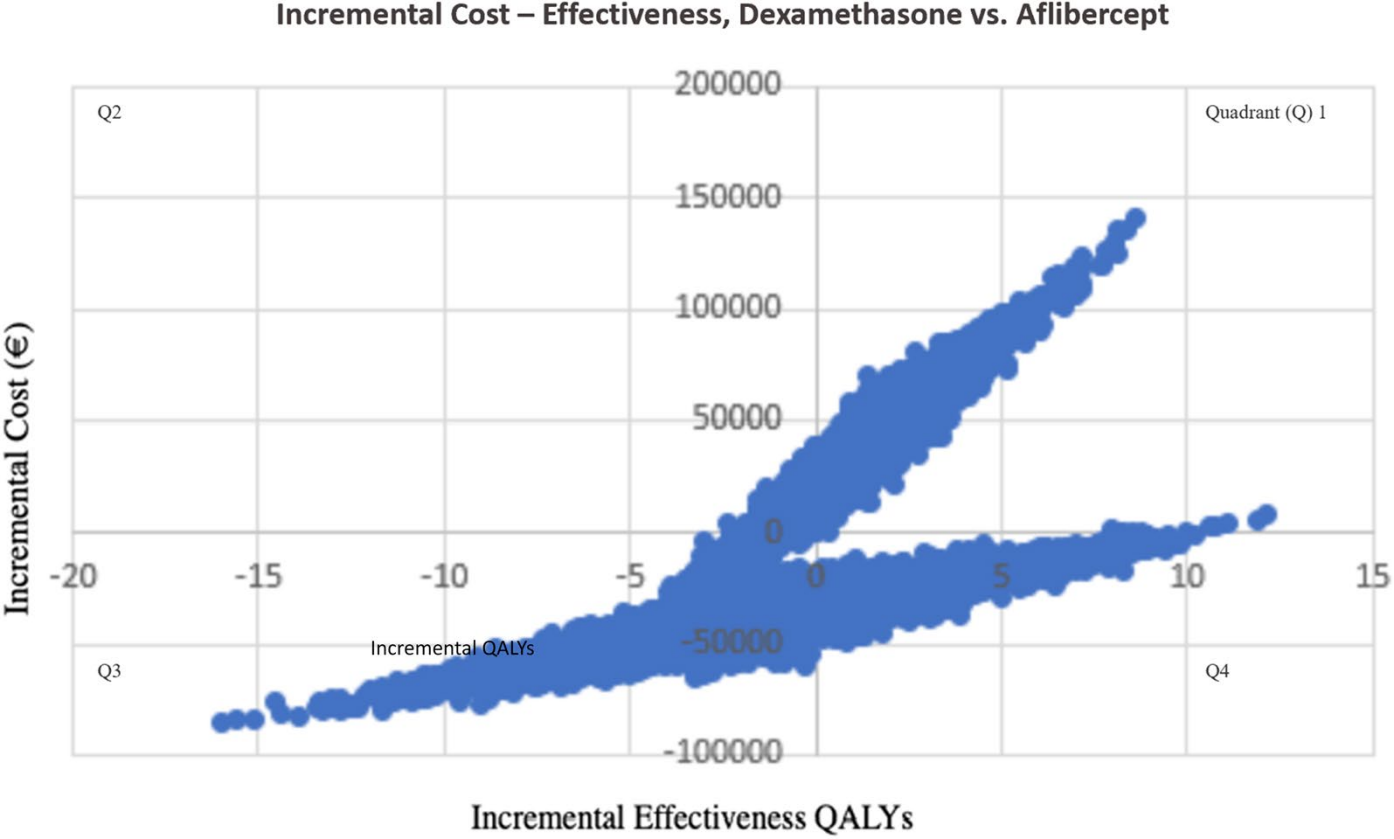
ICER scatterplot generated by MonteCarlo simulation. The ICER is €29,002/QALY. The model has been run 10,000 times. In 35% of cases dexamethasone is more expensive and provides more QALYs than aflibercept and in 31% of cases, dexamethasone is less expensive and provides less QALYs than aflibercept. In quadrant II it is showed that in 8% of cases dexamethasone is more expensive and provides less QALYs than aflibercept and in quadrant IV 26% of cases resulted, which means that dexamethasone is less expensive but provides more QALYs

**Fig. 4:**
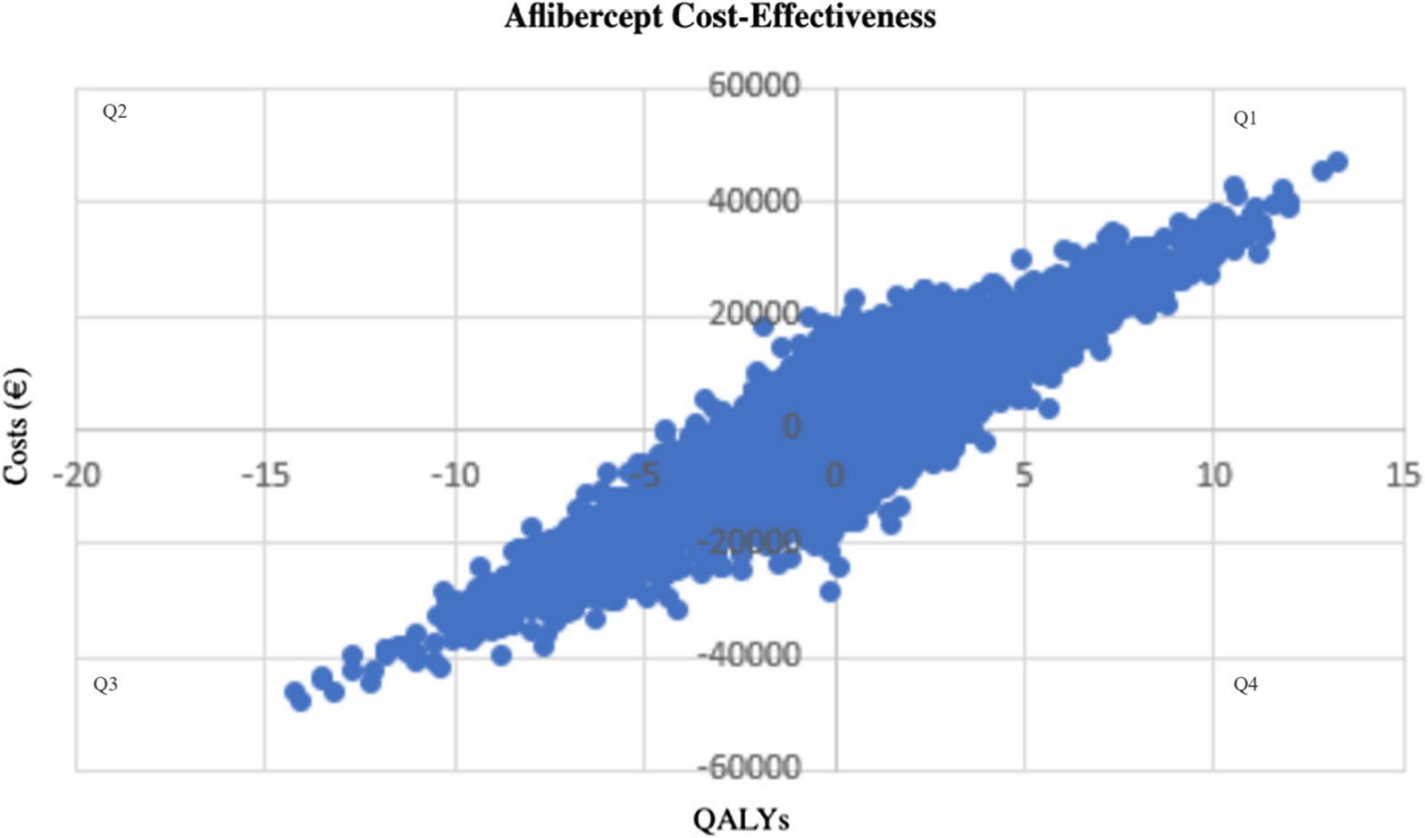
Cost-effectiveness scatterplot for aflibercept generated by MonteCarlo simulation. In quadrant I 46% of cases resulted, which means that the greater the cost, the greater the quality of life obtained. In quadrant II it is showed that in 11% of cases, the higher the cost, the fewer QALYs are obtained. In quadrant III 37% of cases resulted, which means that the fewer the cost, the fewer the quality of life obtained and 6% of cases resulted in quadrant IV, which means that the fewer the cost, the greater QALYs are obtained

**Fig. 5:**
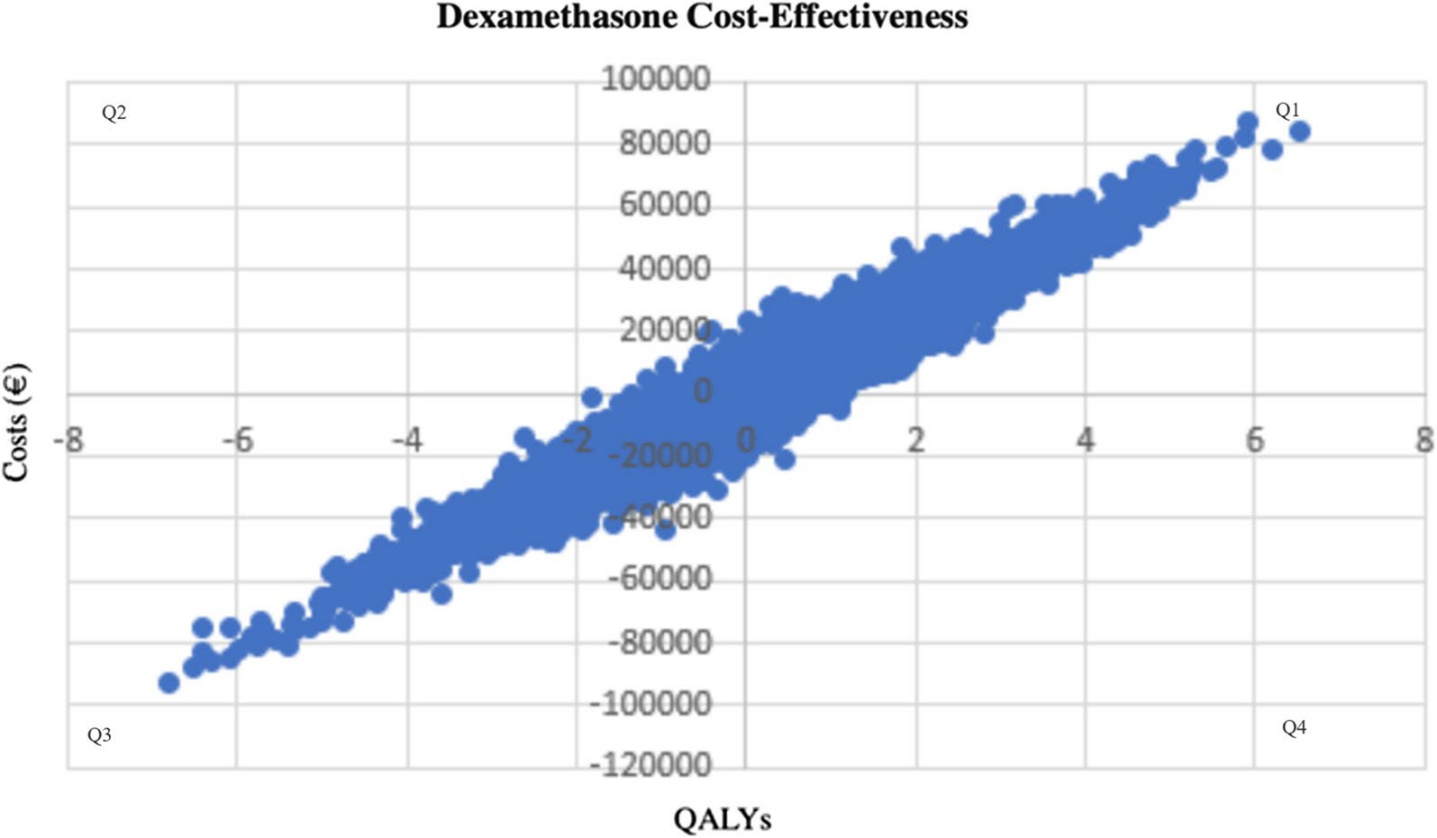
Cost-effectiveness scatterplot for dexamethasone generated by MonteCarlo simulation. In quadrant I 47% of cases resulted, which means that the greater the cost, the greater the quality of life obtained. In quadrant II it is showed that in 8% of cases, the higher the cost, the fewer QALYs are obtained. In quadrant III 36% of cases resulted, which means that the fewer the cost, the fewer the quality of life obtained and 9% of cases resulted in quadrant IV, which means that the fewer the cost, the greater QALYs are obtained

We performed a MonteCarlo simulation (Fig. 3) to study the degree of uncertainty of our ICER variable, as well as an independent simulation to assess the uncertainty of the cost-effectiveness result for each of the two drugs studied (Figs. 4 and 5).

MonteCarlo simulation or MonteCarlo method predicts a set of results based on an estimated range of values against a set of fixed input values. In other words, a MonteCarlo simulation creates a model of possible outcomes by taking advantage of a probability distribution, such as a uniform or normal distribution, for any variable that has inherent uncertainty.

Since the ICER is the ratio between the incremental cost and the incremental QALY, these are our two variables to consider.

In our study we consider the results obtained in 134 patients. Given that DM-2 has a prevalence of approximately 12% in our environment, of which 5.5% develop DME and that the Zaragoza III Health Sector has a population of 267.525 inhabitants, we decided to apply the MonteCarlo method to generate 10,000 random repetitions of incremental cost and incremental QALY data pairs with their corresponding standard deviation.

As shown in Fig. 3, the results we obtained show that most of the points are in quadrants I and III, which means that in 35% of cases dexamethasone is more expensive and provides more QALYs than aflibercept and in 31% of cases, dexamethasone is less expensive and provides less QALYs than aflibercept respectively. In quadrant II it is showed that in 8% of cases dexamethasone is more expensive and provides less QALYs than aflibercept and in quadrant IV are located the 26% of cases, which means that dexamethasone is less expensive but provides more QALYs.

Figures 4 and 5 show that the higher the cost, the higher the quality of life obtained, with the Person coefficient being higher in the case of dexamethasone. This means that for a fixed cost X, a quality of life or QALY with a lower margin of error can be expected; it can be made a more reliable prediction than with aflibercept.

**Fig. 6:**
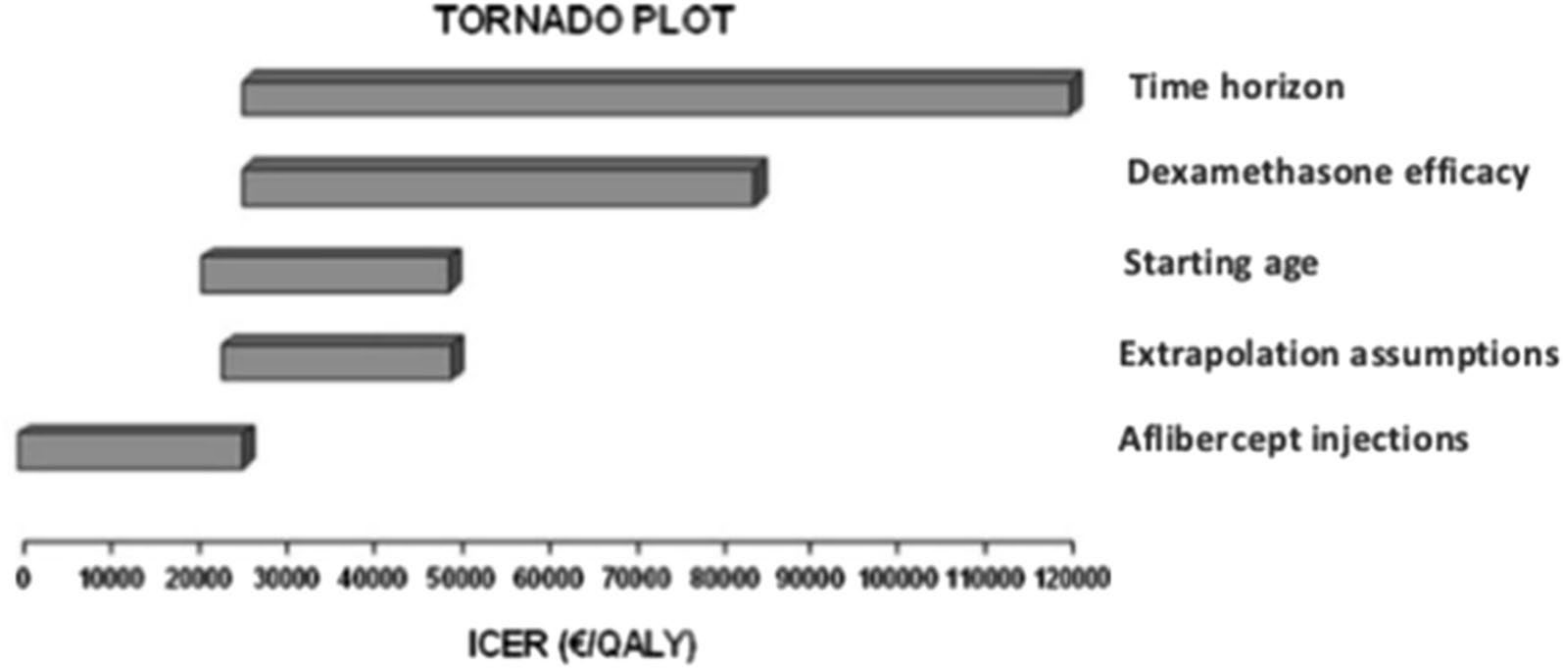
Tornado plot. The time horizon and the chosen extrapolation method, the source for dexamethasone efficacy, and the number of aflibercept injections are the key model drivers, with greater impact on the cost-effectiveness results

We also provided a tornado plot (Fig. 6) to allow the reader to intuitively assess those factors with greater impact in the incremental cost-effectiveness ratio.

## Discussion

In the current environment, this analysis provides the first evidence on the cost-effectiveness of treatment for DME that causes visual impairment in our region.

The dilemma of whether to adopt a new drug has a simple answer if it produces more health gain than the competing alternatives at a lower cost. However, if the new drug is more effective but more expensive than the competing alternatives, clinicians and decision makers have to study whether the new drug provides “good value for money”. Although these thresholds have been criticized [19], published economic evaluations generally use them to tag a new drug as “cost-effective” or “not cost-effective” [20]. These thresholds implicitly represent the health gain forgone when one drug is discarded in favor of the competing intervention, in other words, the opportunity cost of choosing a drug over another. Our study confirms the efficacy and safety of both alternatives for the treatment of DME in Spanish patients. The results confirm that the benefits of aflibercept 40 mg/ml solution for injection (Eylea®) and dexamethasone delayed-release device 700 μg in an applicator (Ozurdex®) are similar in Spanish patients in the Zaragoza III health sector compared to the general populations of the previously carried out phase 3 VIVID and VISTA studies [9]. Our analyses show that the cost per QALY gained with dexamethasone in a lifetime horizon is €29,002, just below the €30,000/ QALY threshold recommended [21] in Spain.

This outcome, however, is sensitive to alternative scenarios explored in the sensitivity analyses. Sensitivity analyses are used to explore the impact of alternative scenarios and uncertainty in model parameters on the cost-effectiveness results. We handled parameter uncertainty by probabilistic sensitivity analysis [19]. According to this analysis, dexamethasone was the therapy of choice in 54% of cases below the threshold of €30,000/QALY in the lifetime horizon (Fig. 2). In addition to probabilistic sensitivity analysis, we performed several univariant and bivariant sensitivity analyses relevant to the clinician. According to these analyses, the factor with greatest impact on the cost-effectiveness results is the chosen time horizon (Fig. 6). We obtained an ICER of €107,159/QALY in a one-year time horizon, which is the duration of our study. Duration of dexamethasone therapy is not restricted to one year. Thus, costs and outcomes over a lifetime horizon were analyzed. Several approaches for extrapolating data from clinical trials over the lifetime horizon were explored. In the reference case the ‘continuous treatment effect’ approach was tested, under which both treatments were administered, with efficacy lasting for both, over the entire time horizon [11].

A dosing strategy similar to that used in the PrONTO trial using ranibizumab [22] has been adopted by ophthalmologists in routine clinical practice [23]. In the PrONTO trial, after a loading phase of three consecutive monthly injections, subsequent injections were performed on an “as needed” basis based on monthly OCT assessment. The PrONTO trial [22] is a well-conducted prospective study, but lack of randomization and comparison with a control group make the results less reliable. However, an ICER of €4623/QALY is obtained.

The impact of the degree of initial VA loss on the cost-effectiveness results was explored. Compared to the reference case, the cost per QALY rose when the cohort of patients started with a VA better than 20/40, probably because patients cannot improve their vision from this state. When the cohort started at the lower VA state (VA ≤ 20/400), ICERs rose as well, probably because fewer patients were able to reach those states with better VA and utility values. Initial VA states with corresponding ICERs are shown in Table 5.

The method for utility elicitation had an impact on the cost-effectiveness results. In the study by Brown et al. [24] from which we took utilities, patients’ preferences for the same VA state were higher when obtained by the standard gamble method compared to the time trade off method. How the choice of one method over the other influenced the cost-effectiveness results was explored, and greater ICERs with utilities obtained by the standard gamble method were found.

### Study strengths and limitations

A strength of our study that is worth highlighting is that it is a prospective, population-based study, representative of the Spanish population and representative of the standard management of patients with diabetic retinopathy.

A limitation of the study is that it was conducted from the second-eye, or better-seeing eye perspective. In this frame, both treatments were applied to the eye which has the greatest impact on the patients’ quality of life [25]. If treatments are applied to the worst-seeing eye, overall VA may not improve in many cases. This second-eye perspective, however, is common in clinical practice. DME develops in the contralateral eye in ≥ 87% of patients with diabetes over 5 years if ≥ 4 risk factors are present.

Moreover, vision loss in the first eye may be secondary to diseases other than diabetes. We believe this approach does not bias results, because the better-case scenario is applied to both aflibercept and dexamethasone.

Another possible limiting factor could be the fact that data obtained from other published articles have been used in which the patients studied could have different baseline characteristics compared to our patients. However, after conducting an analysis comparing the main baseline characteristics (mean age, median age, sex, race and educational level) of the reference population versus the study population, we did not find significant differences for any of the aforementioned characteristics. In the study by Brown et al. [16] from which we took utilities, mean age was 63 years with a range of 28 to 87 years, and a median age of 64 years in their study, versus a mean age of 68.1 years in our study with a range of 32 to 90 years and a median age of 69 years. Patients from Canada, France, Germany, Spain and the United Kingdom participated in the study by Brown et al*.* [16]. Demographic and clinical characteristics of the surveyed population were similar among countries and Caucasians represented 97.7% to 100% of the patients.

Given the age of the study population, recall bias may have led to under-reporting of medical resource utilization. Additionally, this study did not include nursing-home patients. As a result, costs derived from VA loss may be underestimated. If all these costs were taken into account, the disease burden would increase, and a more favorable result for dexamethasone (i.e. a lower ICER), would be obtained.

Randomized controlled trials comparing ranibizumab and aflibercept are ongoing. Raftery et al*.* found that the efficacy of ranibizumab in relation to aflibercept would have to be around 2.5 times greater to be regarded as a cost-effective strategy [25].

## Conclusions

In the present study, we examined the cost-effectiveness of dexamethasone compared to aflibercept in the naïve DME population from the societal perspective. In a lifetime horizon, we obtained an ICER of €29,002/QALY, which is just below the €30,000/QALY threshold recommended [21] in Spain. This ICER was sensitive to alternative scenarios, mainly the selected time horizon and the chosen extrapolation method, the source for dexamethasone efficacy and the number of aflibercept injections.

## Data Availability

All data generated or analyzed during this study are included in this published article.

## Abbreviations

DME: Diabetic macular edema
VA: Visual acuity
QALYs: Quality-Adjusted Life Years
ICER: Incremental cost-effectiveness ratios
SD: Standard deviation
HUI: Health Utilities Index
VFQ-25: Visual Function Questionnaire
